# Small Extracellular Vesicles with a High Sphingomyelin Content Isolated from Hypertensive Diabetic db/db Mice Inhibits Calcium Mobilization and Augments Amiloride-Sensitive Epithelial Sodium Channel Activity

**DOI:** 10.3390/biology14030252

**Published:** 2025-03-01

**Authors:** Hunter Ramsay, Ling Yu, Faisal F. Alousi, Abdel A. Alli

**Affiliations:** 1 Department of Medicine, Division of Nephrology, Hypertension, and Renal Transplantation, University of Florida College of Medicine, Gainesville, FL 32610, USAlingyu@ufl.edu (L.Y.); f.alousi@ufl.edu (F.F.A.); 2 Department of Physiology and Aging, University of Florida College of Medicine, Gainesville, FL 32610, USA; 3 Department of Physiology, College of Medicine, Imam Abdulrahman Bin Faisal University, Dammam 31441, Saudi Arabia

**Keywords:** extracellular vesicles, hypertension, diabetes, calcium, sphingomyelin

## Abstract

Extracellular vesicles (EVs) contain bioactive lipids that play a key role in pathophysiology. We hypothesized that EVs released from salt-loaded hypertensive diabetic db/db mice have increased bioactive lipid content that inhibits intracellular calcium mobilization and increases the activity of renal epithelial sodium channels (ENaC). An enrichment of sphingomyelins (SMs) was found in small urinary EVs (uEVs) isolated from salt-loaded hypertensive diabetic db/db mice (*n* = 4) compared to non-salt loaded db/db mice with diabetes alone (*n* = 4). Both groups of mice were included in the same cohort to control for variability. Cultured mouse cortical collecting duct (mpkCCD) cells loaded with a calcium reporter dye and challenged with small uEVs from hypertensive diabetic db/db mice showed a decrease in calcium mobilization when compared to cells treated with small uEVs from diabetic db/db mice. The amiloride-sensitive transepithelial current was increased in mpkCCD cells treated with small uEVs with abundant sphingomyelin content from hypertensive diabetic db/db mice in a dose- and time-dependent manner. Similar results were observed in mpkCCD cells and *Xenopus* 2F3 cells treated with exogenous sphingomyelin in a time-dependent manner. Single-channel patch clamp studies showed a decrease in ENaC activity in cells transiently transfected with sphingomyelin synthase 1/2 specific siRNA compared to non-targeting siRNA. These data suggest EVs with high sphingomyelin content positively regulate renal ENaC activity in a mechanism involving an inhibition of calcium mobilization.

## Simple Summary

The epithelial sodium channel (ENaC) expressed in the kidney plays an important role in maintaining total body salt balance and blood pressure regulation. The activity of the epithelial sodium channel is positively regulated by the availability of anionic lipids at the plasma membrane and negatively regulated by increases in intracellular calcium levels. Here, we investigated regulation of the epithelial sodium channel by the lipid sphingomyelin (SM) in small extracellular vesicles isolated from the urine of mice with high blood pressure and diabetes compared to mice with diabetes alone. Activity of the epithelial sodium channel was measured in two different kidney cell types after the cells were treated with small extracellular vesicles with high sphingomyelin content from the two groups of mice and after the knockdown of enzymes that generate sphingomyelin. In this study, we show increased sphingomyelin content in small extracellular vesicles from mice with both high blood pressure and diabetes increases epithelial sodium channel activity in two different kidney cell types in a time-dependent manner. Knockdown of enzymes that generate sphingomyelin showed similar results. Mechanistically, the inhibition of calcium influx presumably was responsible for the effect of sphingomyelin content in small extracellular vesicles on epithelial sodium channel activity. Taken together, results from this study will provide a better understanding of the regulation of epithelial sodium channel activity in the kidney during the development of high blood pressure secondary to diabetes.

## 1. Introduction

Epithelial sodium channels (ENaC) expressed in the distal tubule and collecting ducts of the kidney play an important role in regulating overall salt balance and blood pressure regulation. In the mouse kidney, these channels function most efficiently as a heterotrimeric complex of alpha, beta, and gamma subunits [1].

Numerous studies have demonstrated a role for various lipids in the regulation of renal ENaC activity. Phosphoinositides are rare but important signaling lipids that regulate ENaC activity. Phosphatidylinositol 4,5-bisphosphate (PI(4,5)P2) [2] and phosphatidylinositol 3,4,5-trisphosphate [3] are known to regulate renal ENaC activity at the luminal/apical plasma membrane. The lipid raft associated protein myristoylated alanine-rich C-kinase substrate (MARCKS)/MARCKS like protein 1 (MLP-1) sequesters anionic phospholipid phosphates and increases the local concentration of these lipids in close proximity to ENaC to allow for positive regulation of channel activity [4]. Sphingomyelin synthase 1 and 2 (SGMS 1/2) are responsible for the production of plasma membrane sphingomyelins [5,6]. Sphingomyelins play an important role in maintaining microdomain fluidity. Different sphingolipids are known to regulate calcium flux by various mechanisms [7]. An increase in intracellular calcium levels is known to negatively regulate the association between ENaC, MARCKS/MARCKS like-protein 1, and the plasma membrane. One study demonstrated an increase in intracellular calcium causes formation of a calcium calmodulin complex that causes MARCKS/MARCKS-like protein 1 to translocate from the plasma membrane to the cytoplasm, subsequently resulting in a decrease in ENaC activity [8]. Extracellular vesicles (EVs), including microvesicles and exosomes, have a high concentration of various molecules including lipids. EVs are released by various cells [9] and taken up by recipient cells [10]. As a result, EVs allow for intercellular communication [11], intracellular signaling [12], and regulation of plasma membrane dynamics [13]. Previous studies from our group showed small urinary extracellular vesicles (uEVs) have a high content of different lipids including sphingomyelins. EVs are known to shuttle cargo including lipids from one cell to another and may also regulate renal ENaC activity.

Multiple studies have implicated a role for sphingomyelins (SMs) in various kidney diseases. A recent study reported sphingomyelin contributes to the initiation and progression of kidney disease [14]. Granata et al. reported the involvement of sphingomyelin in the pathogenesis of medullary sponge kidney (MSK) disease [15]. Another study showed sphingomyelin accumulation is associated with the development of renal cancer [16]. Results from a study by Miyamoto et al. showed SM(d18:1/16:0) causes activation of the glycolytic pathway in mesangial cells in vitro and may play a role in the pathophysiology of obesity and diabetes [17].

In this study, we utilized the db/db mouse model which develop hypertension upon salt-loading [18] and aging [19], while C57Bl6 mice are generally resistant to developing hypertension from salt loading. Another reason for using the db/db mouse model is that these mice have polyuria [20] and this allows us to collect a desirable amount of urine for isolation of uEVs.

The sphingomyelin-ceramide pathway has been shown to play a role in the pathogenesis of diseases associated with diabetes, but a direct mechanistic role for sphingomyelins in the pathophysiology of these complications including diabetes-associated hypertension is not understood. Although renal ENaC is known to be regulated by various lipids within the plasma membrane and by EVs, the regulation of ENaC by EVs with high sphingomyelin content and an increase in sphingomyelin levels in cell membranes has not been studied. Here, we performed mechanistic studies to test our hypothesis that sphingomyelin positively regulates ENaC activity in distal tubule cells and in collecting duct cells.

## 2. Materials and Methods

### 2.1. Cell Culture

*Xenopus* 2f3 distal tubule cells (originally obtained by Dr. Benos) were grown in culture on permeable supports and maintained in Dulbecco’s modified Eagle’s medium/Ham’s F-12 base media (Invitrogen, Carlsbad, CA, USA) that was supplemented with 1.5 μm aldosterone (Millipore Sigma; St. Louis, MO, USA), 0.1% penicillin-streptomycin solution (ThermoFisher Scientific, Waltham, MA, USA), and 5% fetal bovine serum (Corning; Glendale, AZ, USA). *Xenopus* 2f3 cells were maintained in an incubator calibrated at 4% CO_2_ and 26 °C. The media was changed out every 3–4 days. Only *Xenopus* 2f3 cells with a passage number of less than 12 were used for experiments.

Mouse mpkCCD cells (originally obtained from Dr. Alain Vandewalle (Institut National de la Santé et de la Recherche Médicale Unité; France) were cultured in a 1:1 mixture of DMEM and Ham’s F-12 medium (GIBCO; Grand Island, NY, USA) supplemented with 1 nM triiodothyronine, 50 nM dexamethasone, 20 mM HEPES, 2 mM l-glutamine, 0.1% penicillin-streptomycin solution (ThermoFisher Scientific), and 2% heat-inactivated FBS (Corning). mpkCCD cells were maintained in an incubator calibrated at 5% CO_2_ and 37 °C. The media was changed out every 3–4 days. Only mpkCCD cells with a passage number of less than 14 were used for experiments.

For both cell lines, a fluorescence-based mycoplasma detection kit (ThermoScientific) was used to routinely check for contamination when performing cell culture work.

### 2.2. Animal Studies

Ten-week-old diabetic db/db mice were purchased commercially (Jackson Laboratory, Bar Harbor, ME, USA) and individually housed in metabolic cages. The mice were on a 12-h light–dark cycle and housed in an environment of approximately 24 °C and 70% relative humidity. Since our group previously showed C57Bl6 wild-type mice are resistant to developing hypertension from salt-loading and db/db mice develop hypertension from 8 days of salt-loading, we utilized two groups of db/db mice in this study [18]. One group of db/db mice were maintained on a normal salt diet (0.4% NaCl; Teklad Rodent diet (Envigo Inc., Indianapolis, IN, USA) and another group of db/db mice were maintained on a high salt diet (4% NaCl; a Teklad Rodent diet (Envigo Inc.) for 10 days. Urine was collected daily. All animal studies were performed under an approved University of Florida, Gainesville, FL Institutional Animal Care and Use Committee (IACUC) protocol (Protocol Number 202011157).

### 2.3. uEV Isolation

A total of 30 mL of urine was subjected to centrifugation at 1000× *g* for 10 min, followed by filtration using a 0.2 μm Nalgene filters (Thermo Fisher Scientific). To remove large extracellular vesicles including microvesicles and apoptotic bodies greater than 200 nm in size, we performed ultracentrifugation at 118,000× *g* for 70 min at 4 °C using a fixed-angle rotor Ti-70 (Beckman Coulter, Inc., Brea, CA, USA). The resulting pellet was resuspended in ultrapure 1X PBS. The uEVs were assessed for purity and size confirmation by nanoparticle tracking analysis, transmission electron microscopy, and Western blotting for common uEV markers.

### 2.4. Nanoparticle Tracking Analysis

A NanoSight NS300 machine (Malvern Instruments, Malvern, UK) was used to characterize each uEV preparation. The samples were analyzed using Nanoparticle Tracking Analysis (NTA) 3.2 Build 16 software.

### 2.5. Transmission Electron Microscopy Analysis of uEVs

A pooled sample (*n* = 4) of uEVs from diabetic db/db mice and a pool sample (*n* = 4) from hypertensive diabetic db/db mice were prepared in a paraformaldehyde solution (2% final concentration with 1XPBS). Next, 10 µL of the prepared uEV samples were incubated with Formvar-carbon coated grids (Ladd Research Industries, Williston, VT, USA) for 20 min at room temperature. Thereafter, 100 μL of 1 × PBS was pipetted onto the grids before being incubated for 3 min at room temperature. The grids were then transferred to a 50 μL solution of glutaraldehyde (Ladd Research Industries) (1% in 1X PBS) and left to incubate for 5 min at room temperature. Next, the grids were subject to eight 2 min washes in 50 μL of 1X PBS. The grids were then incubated in 50 μL of a uranyl-oxalate solution, pH 7 prepared from uranyl acetate (Ladd Research Industries) and oxalic acid (Sigma-Aldrich). Afterward, the grids were placed in 50 μL of a methyl cellulose-uranyl-acetate solution and incubated for 10 min on a cold plate. A stainless-steel loop was used to carefully remove the grids that were then blotted on the side using No. 1 Whatman filter paper. After being allowed to air dry for 5 min, the grids were viewed on a Hitachi H-7600 transmission electron microscope (Hitachi High Technologies America, Inc., Clarksburg, MD, USA) with AMT image capture software version 600.335p (Advanced Microscopy Techniques, Danvers, MA, USA) 

### 2.6. Patch Clamping

The cell-attached configuration was used in all patch-clamp experiments. The resistances of the pipettes were between 8 and 10 MΩ when filled with and immersed in a patch solution containing (in mM) 96 NaCl, 3.4 KCl, 0.8MgCl2, 0.8 CaCl2, and 10 HEPES, with pH adjusted to 7.4 by NaOH for 2F3 cells. While the patch and bath solution for mpkCCD cells contained (in mM) 150 mm NaCl, 1 mm CaCl2, 2 mm MgCl2, 10 mm HEPES (pH 7.4). Single channel recordings were made from individual cells for ~8–10 minat pipette holding potentials of 0 or 20 mV. The software used for data acquisition was Clampex 10.7 and the software used for data analysis was Clampfit 10.7.

### 2.7. Transient Transfection of siRNA

mpkCCD cells were transfected with sphingomyelin synthase 1 and 2 siRNA SMARTpool or siGENOME Non-Targeting siRNA SMARTpool (Cat. No. D-001206-14-05) (Horizon Discovery Biosciences, Cambridge, UK) as a control using DharmaFECT 2 transfection reagent (Cat No. T-2002-02) (Horizon Discovery Biosciences) according to the manufacturer’s instructions. Transfection efficiency was assessed by measuring knockdown at the protein level by Western blotting.

### 2.8. SDS PAGE and Western Blotting

A total of 20 µg total protein from uEV lysates prepared in 1X RIPA (ThermoScientific) was resolved on 4–20% gradient gels (ThermoScientific) for 1 h using a Criterion electrophoresis system (Bio-Rad, Hercules, CA, USA). The proteins were transferred to nitrocellulose membranes (ThermoScientific) using a Criterion transfer system (BioRad). The blots were blocked in 5% nonfat dry milk for 1 h and then incubated in 1:1000 dilution of primary antibody (Table 1) prepared in 5% 1X TBS 5% BSA solution overnight at 4 °C. Next, the blots were washed with 1X TBS and then incubated with a 1:3000 dilution of secondary antibody (Bio-Rad) prepared in a blocking solution. The blots were then washed with 1X TBS and incubated for 7 min in ECL reagent (Bio-Rad) before being developed on an imager (Bio-Rad).

### 2.9. Transepithelial Measurements

mpkCCD cells and *Xenopus* 2F3 cells were grown on transwell-permeable supports (Corning) to confluency. After 7–10 days in culture, transepithelial voltage and transepithelial resistance across cell monolayers were measured using an epithelial volt-ohmmeter (World Precision Instruments, Sarasota, FL, USA) equipped with chopstick electrodes. The equivalent short-circuit current was calculated after measuring transepithelial voltage and transepithelial resistance according to Ohm’s law formula, V = I × R, where V represents voltage, I represents current, and R represents resistance.

### 2.10. Microscopy

Calcium mobilization was measured by first loading mpkCCD cells for 30 min at room temperature with 0.5 µM of the fluorogenic calcium-sensitive dye Cal-520AM (AAT Bioquest, Pleasanton, CA, USA) Catalog No. 21130) diluted with Ca^2+^-free Hanks’ balanced salt solution (HBSS) (Gibco, Thermo Fisher Scientific) (Catalog number 14175-095). The cells were stimulated with 1 µM with the calcium-mobilizing drug, ionomycin (Invitrogen, Thermo Fisher Scientific) (Catalog number I24222). Brightfield images were taken using a Zyla monochrome camera with a 40× magnification immediately before and after fluorescent imaging.

### 2.11. Measurement of Sphingomyelins

EVs were lysed by sonication in RIPA buffer and the amount of sphingomyelins present in the samples was determined by performing an in vitro sphingomyelin assay (Catalog Number STA-601) (Cell Biolabs Inc., San Diego, CA). Briefly, uEVs lysates were prepared at a 1:50 dilution with assay buffer and a series of eight sphingomyelin standards were prepared by serial dilution. Next, 10 µL of sphingomyelin standards or samples were added to a 96-well plate before 100 µL of reaction reagent was added and mixed. The plate was incubated for 60 min at 37 °C while being protected from the light. The plate was read for fluorescence excitation of 570 nm and emission of 590 nm and the concentration of sphingomyelin within the samples (in relative fluorescence units) was determined from the sphingomyelin standard curve.

### 2.12. Statistical Analysis

SigmaPlot software version 15.0 (Jandel Scientific, San Rafael, CA, USA) was used to perform statistical analyses between groups and to plot the data. Differences between groups were evaluated by performing a Student *t* test. Statistical significance between the groups was defined as a *p*-value < 0.05.

## 3. Results

### 3.1. Increased Release of Small uEVs in Salt-Loaded Hypertensive Diabetic db/db Mice

Previous studies from our group showed diabetic db/db mice develop hypertension after salt loading [18,21]. In addition, we and others showed these salt-loaded hypertensive diabetic db/db mice have polyuria [18,20,21,22]. The cohort of mice maintained on a normal salt diet and high salt diet were both 8–10 weeks of age and there was no significant difference in the body weights between the two groups (40.9 ± 1.7 g for the db/db mice on a normal salt diet and 48.1 ± 2.9 g for the db/db mice maintained on a high salt diet). First, each uEV preparation from each group was characterized by nanoparticle tracking analysis. To determine whether salt-loaded hypertensive mice release more EVs into the urine, a small uEV concentration was measured from urine samples from four mice in each of the two groups. There was a greater concentration of small uEVs from the salt-loaded (HS) hypertensive diabetic db/db mice compared to the db/db mice with diabetes alone (NS) (Supplementary Figure S1A). The size between the two groups was comparable (Supplementary Figure S1A). Next, Western blotting for the uEV markers CD9, caveolin-1, flotillin, and syntenin showed the presence of each uEV marker in each uEV preparation from the two groups (Supplementary Figure S1B–E). Nevertheless, there was no appreciable difference in any of these uEV markers between the two groups. Transmission electron microscopy analysis was performed to assess the purity of the uEVs from each group. The majority of the uEVs were less than 50 nm in diameter and there were no appreciable levels of aggregated proteins or large vesicles including apoptotic bodies (Supplementary Figure S1F).

### 3.2. Enrichment of Sphingomyelins in Small uEVs from Salt-Loaded Hypertensive Diabetic db/db Mice Compared to Diabetic db/db Mice

**Figure 1:**
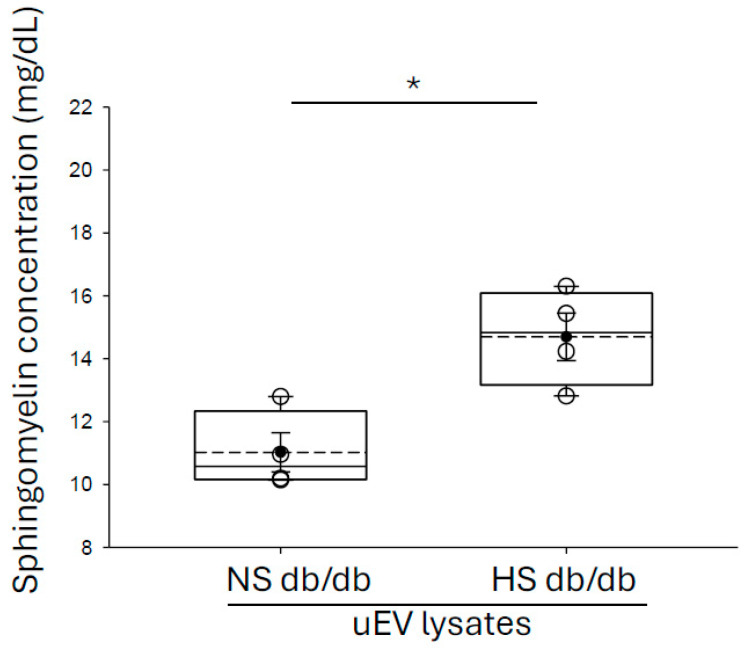
Quantification of sphingomyelin concentration in small urinary extracellular vesicles isolated from the urine of diabetic db/db mice and salt-loaded hypertensive diabetic db/db mice. EVs were isolated from separate collections of urine (30 mLs each) from diabetic db/db mice (N = 4) and salt-loaded hypertensive diabetic db/db mice (N = 4). The concentration of each uEV preparation was normalized after performing NanoSight analysis. The small uEVs were lysed in RIPA buffer and the number of sphingomyelins present in the samples was determined by performing an in vitro sphinomyelin fluorescent-based assay. N = 4 per group. A Student *t* test was performed to compare the two groups. * represents a *p*-value < 0.05.

A previous study by Dorrance et al. showed sphingomyelin (SM) concentration is greater in membranes of stroke-prone spontaneously hypertensive rats compared to Wistar–Kyoto rats [23]. Here, we investigated for the first time whether small uEVs from salt-loaded hypertensive diabetic db/db mice have greater sphingomyelin content compared to diabetic db/db mice. As shown in Figure 1, the concentration of sphingomyelin was greater in small uEVs from the hypertensive diabetic group compared to db/db mice with diabetes alone.

### 3.3. Increased Sphingomyelins Content in Small uEVs from Salt-Loaded Hypertensive Diabetic db/db Mice Inhibit Calcium Mobilization in mpkCCD Cells

**Figure 2:**
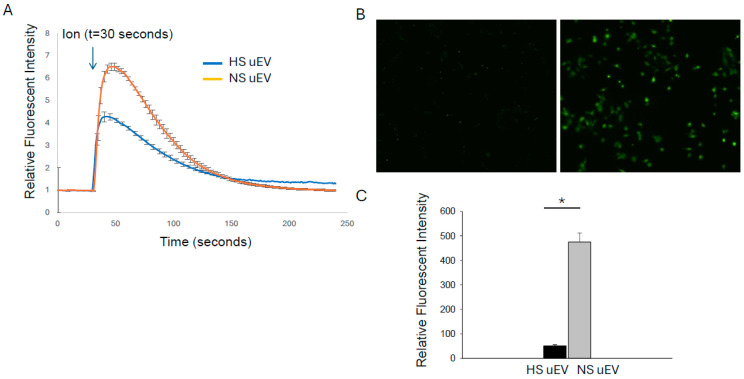
Small urinary EVs from salt-loaded hypertensive diabetic db/db mice compared to diabetic db/db mice inhibit calcium influx in mpkCCD cells. (**A**) Summary analysis showing a transient influx in intracellular calcium mediated by ionomycin (Ion) after pretreating mpkCCD cells with small uEVs from each group; (**B**) Representative immunofluorescence images before (left panel) and after (right panel) calcium influx; (**C**) Summary graph of the relative fluorescent intensity in (**B**). N = 4 per group. A Student *t* test was performed to compare the two groups. uEVs refer to urinary extracellular vesicles. NS refers to normal salt (non-salt loaded diabetic db/db mice). HS refers to high salt (salt-loaded hypertensive diabetic db/db mice). * represents a *p*-value < 0.05.

The activity of renal ENaC is known to be inhibited by calcium-dependent mechanisms. Therefore, we investigated whether small uEVs from salt-loaded hypertensive diabetic db/db mice compared to small uEVs from db/db mice with diabetes alone alter intracellular calcium mobilization in mpkCCD cells. There was a significant decrease in intracellular calcium mobilization in mpkCCD cells challenged with small uEVs from the salt-loaded hypertensive diabetic db/db mice compared to cells challenged with small uEVs from db/db mice with diabetes alone (Figure 2).

### 3.4. Exogenous Sphingomyelin Augments Amiloride-Sensitive Transepithelial Current in Mouse mpkCCD Cells and in Xenopus 2f3 Cells

**Figure 3:**
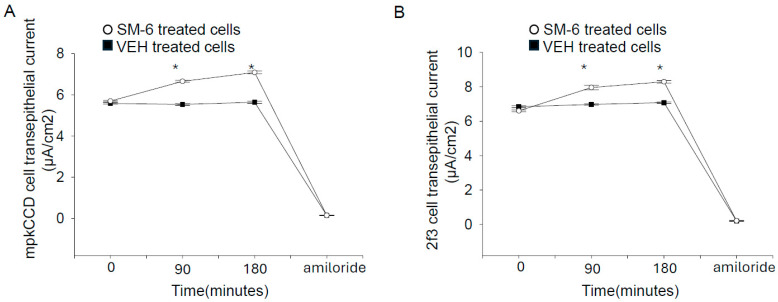
Exogenous sphingomyelins (SM-6) (1 μM) augment amiloride-sensitive transepithelial current in mpkCCD cells and in *Xenopus* 2f3 cells. (**A**) transepithelial current in mpkCCD cells treated with SM-6 or VEH. (**B**) transepithelial current in 2f3 cells treated with SM-6 or VEH. At the end of the experiment for each cell type, 1 μM amiloride was administered on the apical side as a measure of amiloride-sensitive transepithelial current. Closed squares represent cells treated with vehicle and open circles represent cells treated with SM-6. N = 4 per group. A Student *t* test was performed to make comparisons between the two groups. VEH represents vehicle. SM-6 represents sphingomyelin-6. * represents a *p*-value < 0.05.

To determine whether ENaC activity is enhanced in confluent monolayers, we treated two different cell types with exogenous sphingomyelin and measured transepithelial resistance and voltages before calculating the amiloride-sensitive transepithelial current. Since exogenous SM treatment has been shown to cause cell death in renal tubular cells [24], we treated our cells with a low 1 μM concentration of sphingomyelin for 90 min before measuring the transepithelial current. In addition, in parallel studies we measured cell viability between the groups. Compared to the vehicle, a 1 μM concentration of sphingomyelin for 90 min did not cause an increase in cell viability in either mpkCCD cells or *Xenopus* 2f3 cells. As shown in Figure 3A, the amiloride-sensitive transepithelial current was augmented after treating mpkCCD cells with 1 μM exogenous sphingomyelin (SM-6) for 90 min. Similarly, the same trend was observed in *Xenopus* 2f3 cells treated with the same concentration of SM-6 for 90 min (Figure 3B).

### 3.5. ENaC Protein Expression Is Comparable in mpkCCD Cells Treated with uEVs from Salt-Loaded and Non-Salt Loaded db/db Mice

Western blotting using a specific and validated antibody against ENaC alpha protein showed no appreciable change in protein expression for the uncleaved or cleaved forms of ENaC alpha protein after mpkCCD cells were challenged with small uEVs from either the hypertensive diabetic db/db mice or db/db mice with diabetes alone (Supplementary Figure S2).

### 3.6. siRNA Mediated Knockdown of Sphingomyelin Synthase 1 and 2 Attenuates ENaC Activity in mpkCCD Cells

**Figure 4:**
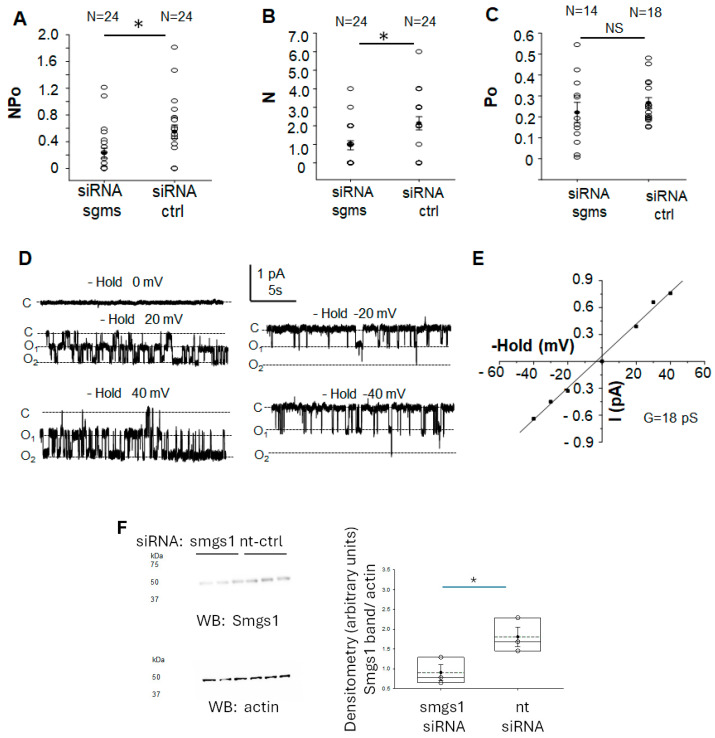
Patch clamp analysis of a highly selective cation channel in mouse mpkCCD cells transiently transfected with sphingomyelin synthase 1 and 2 specific siRNA or non-targeting control siRNA. mpkCCD cells transfected with sphingomyelin synthase 1 and 2 siRNA or non-targeting control siRNA were patched for ENaC single channel activity. (**A**) NPo as a measure of ENaC activity, (**B**) N, as a measure of the number of channels in a patch, (**C**) Po, as a measure of the open probability of the channel. The numbers at the top of the plots indicate the number of patches per group. (**D**) representative traces where C represents the closed state and O represents the open state. (**E**) Current voltage curve. (**F**) Representative Western blot for smgs1 (top blot) to assess siRNA mediated knockdown of Smgs1 and transfection efficiency in mpkCCD cells. The Western blot for actin (bottom blot) was used to assess lane loading. Densitometric analysis and plot of the Smgs1 protein band normalized to actin (right). siRNA sgms refers to sphingomyelin synthase 1/2 specific siRNA. siRNA ctrl refers to non-targeting control siRNA. NS refers to no significant difference between the groups. The number of experiments for each group is given at the top of panels A–C. For NPo and N, N = 24 in each group. A Student *t* test was performed to compare the two groups. * represents a *p*-value < 0.05.

To further corroborate the role of sphingomyelins in positively regulating ENaC activity in mpkCCD cells, we transiently transfected siRNA targeting sphingomyelin ½ to knockdown each enzyme and then patched for ENaC activity using the single-channel patch clamp method. As shown in Figure 4A, ENaC activity was significantly lower in mpkCCD cells transiently transfected with sphingomyelin synthase 1 and 2 siRNA compared to cells transiently transfected with non-targeting control siRNA. The decrease in ENaC activity was mainly at the level of N, density of channels at the membrane (Figure 4B). The open probability of ENaC was comparable between the two groups (Figure 4C). Representative ENaC recordings and a current voltage plot are given in Figure 4D and Figure 4E, respectively.

## 4. Discussion

Our data suggest greater sphingomyelin content in small uEVs from hypertensive diabetic db/db mice augments ENaC activity in the distal tubule cells and collecting duct cells after cellular uptake. In addition, our data suggests the mechanism for the positive regulation of ENaC by EVs with high sphingomylelin content is mediated by a decrease in intracellular calcium mobilization.

ENaC is regulated by a myriad of mechanisms including being negatively regulated by calcium-dependent mechanisms. Montgomery et al. showed calcium mobilization decreases the association between ENaC, PIP2, and its adaptor protein myristoylated alanine-rich C kinase substrate (MARCKS)/MLP-1 [25]. Mechanistically, calcium–calmodulin binding to the MARCKS/MLP-1 protein inhibits its filamentous actin crosslinking ability [26]. Interestingly, modulation of actin cytoskeleton dynamics or its reorganization may affect membrane fluidity in various cell types [27,28]. The findings by Montgomery et al. [25] align with the results of this study, which also shows ENaC is positively regulated by an increase in concentration of specific lipids at the plasma membrane. Increased concentrations of signaling lipids such as PIP2 and bioactive lipids such as SM at the plasma membrane positively regulate ENaC activity and presumably its adaptor protein MARCKS/MLP-1.

The apical plasma membrane of polarized epithelial cells has greater sphingomyelin and glycosphingolipid content while the basolateral membranes are abundant in glycerolipids and phosphatidylcholines [29]. Our data show small uEVs from salt-loaded hypertensive db/db mice have greater sphingomyelin content compared to small uEVs from db/db mice with diabetes alone (Figure 1). Importantly, the lipid profile of EVs resembles that of their parent cells and the lipid content of a recipient cell changes after uptake of EVs. In addition, a previous study by our group showed polarized epithelial cells release two types of EVs across the apical plasma membrane and basolateral plasma membrane that each have a distinct lipid profile [30]. In addition, in vitro studies showed unique lipidomic and proteomic profiles of EVs released across the apical plasma membrane from different kidney cell types [11,30].

The association between sphingomyelin and plasma membrane fluidity has been studied by Klein et al. [31]. Importantly, the lipid composition of cell membranes and membrane fluidity are altered in hypertension [23]. Elevated activity levels of the enzyme that converts ceramide from sphingomyelin, sphingomyelinase, was reported in diabetic animals and patients with diabetes [32,33]. Jiang et al. reported inhibition of acidic sphingomyelinase activity mitigated endothelial dysfunction in db/db mice [34]. We showed in our study transient siRNA-mediated knockdown of sphingomyelin synthases 1 and 2 significantly inhibits ENaC activity in mpkCCD cells mainly at the level of the number of channels in a patch or density of ENaC at the membrane. 

There are some limitations of our current study. First, we used a small sample size of N = 4 diabetic db/db mice and N = 4 salt-loaded hypertensive diabetic db/db mice. A larger number of mice in each group would allow for comparisons to be made between males and females and between age groups in future studies. Second, we did not measure ceramide levels or conduct a lipidomics study to investigate which sphingomyelins and ceramides are increased in the membranes of cells treated with small uEVs. A sphingomyelin-signaling pathway is thought to involve hydrolysis of sphingomyelin to liberate ceramide and then sphingosine. The knowledge gap of whether ceramides would have the opposite effect on renal ENaC activity can be mitigated by performing similar studies using engineered EVs where ceramides are enriched. Additionally, we did not investigate downstream effects of treating mpkCCD cells and *Xenopus* 2f3 cells with sphingomyelinase. A study by Zager et al. showed sphingomyelinase causes ATP depletion/Ca^2+^ ionophore injury and iron mediate cell injury in cultured human proximal tubule cells [24].

Future studies will further investigate whether an increased sphingomyelin content in small uEVs from hypertensive diabetic db/db mice alters specific ENaC-regulating pathways including the protein kinase C pathway and the phospholipase C pathway. In addition, membrane fluidity and dynamics of the actin cytoskeleton will be measured in different ENaC expressing cell types after challenging cells with EVs with abundant sphingomyelin content from hypertensive diabetic mice compared to mice with diabetes alone. Although the scope of this study was to investigate whether epithelial sodium channel activity in collecting duct cells is altered by sphingomyelins content in extracellular vesicles released from hypertensive diabetic db/db mice, future studies may investigate whether renal artery stiffness is affected by sphingolipid metabolism.

## 5. Conclusions

Taken together, the results from this study suggest EVs released during the development of hypertension secondary to diabetes have increased sphingomyelin content and these EVs play a role in the upregulation of renal ENaC activity. Presumably, the mechanism involves suppression of calcium mobilization in ENaC-expressing cells after uptake of the EVs.

## Figures and Tables

**Table 1:** List of antibodies used in this study.

Antibody	Size (kDa)	Company	Catalog Number
Anti-CD9	25 kDa	abcam (Waltham, MA, USA)	ab223052
Anti-Caveolin-1	21 kDa	Cell Signaling Tech (Danvers, MA, USA)	3267
Anti-syntenin	32 kDa	abcam	ab19903
Anti-flotillin	47 kDa	abcam	ab41927
Anti-sphingomyeiln synthase	49 kDa	Proteintech (Rosemont, IL, USA)	19050-1-AP
Anti-Beta actin HRP	42 kDa	Sigma	A3854

## Data Availability

Datasets will be provided upon reasonable request after contacting the corresponding author.

## Supplementary Materials

The following supporting information can be downloaded at: https://www.mdpi.com/article/10.3390/biology14030252/s1, Figure S1: Characterization of small extracellular vesicles isolated from the urine of diabetic db/db mice and salt-loaded hypertensive diabetic db/db mice (A) Nanoparticle tracking analysis of small uEVs isolated from urine samples from diabetic db/db mice maintained on a normal salt diet and from urine samples from hypertensive diabetic db/db mice maintained on a high salt diet. (B) Western blot of the uEV marker CD9 from the two groups, (C) Western blot of the uEV marker caveolin-1 from the two groups, (D) Western blot of the uEV marker flotillin from the two groups, (E) Western blot of the uEV marker syntenin from the two groups, (F) transmission electron microscopy analysis of uEVs from a pooled sample (*n* = 4) of uEVs from diabetic db/db mice maintained on a normal salt diet (left) or uEVs from hypertensive diabetic db/db mice maintained on a high salt diet (right). 100,000× direct magnification; Figure S2: Western blot and densitometric analysis of ENaC alpha protein expression in mpkCCD cells treated with small uEVs isolated from db/db mice maintained on a normal salt (NS) or high salt (HS) diet.; Figure S3: Full Western blot and densitometric analysis of CD9 (Figure S1B), Caveolin-1 (Figure S1C), Flotillin (Figure S1D), and syntenin (Figure S1E); Figure S4: Full Western blot and densitometric analysis ofSMGS1 and actin (Figure 4F); Figure S5: Full Western blot and densitometric analysis of ENaC alpha 59 and Actin.
