# Supplementary material for: Small Extracellular Vesicles with a High Sphingomyelin Content Isolated from Hypertensive Diabetic db/db Mice Inhibits Calcium Mobilization and Augments Amiloride-Sensitive Epithelial Sodium Channel Activity
Source: Biology (Basel). 2025 Mar 1;14(3):252. doi: 10.3390/biology14030252 (PMC11939694; doi:10.3390/biology14030252)
Supplement: Supplementary file 1 [file biology-14-00252-s001.zip › biology-3211372-supplementary.pdf]

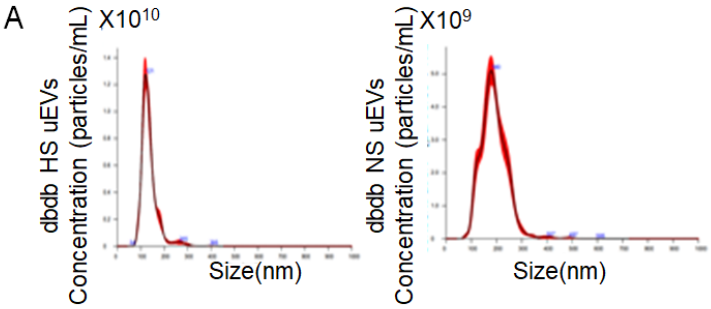

	Size (nm)	Concentration (particles/mL)
db/db NS uEV	168.0	4.19 $X10^{11}$
db/db HS uEV	137.4	1.55 $X10^{12}$
P-value	15.19	0.001

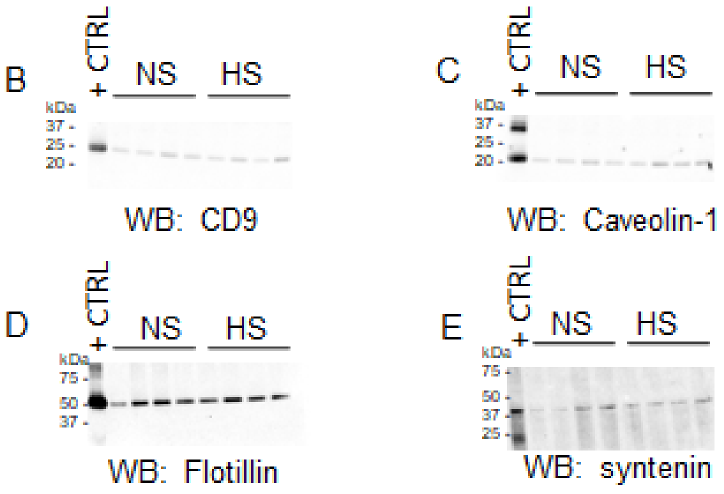

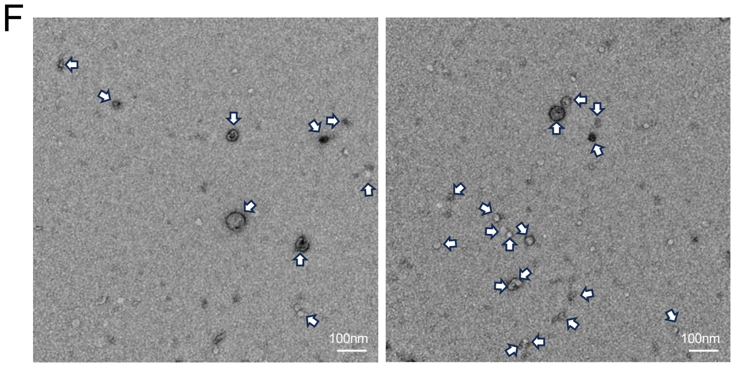

Supplementary Figure 1. Characterization of small extracellular vesicles isolated from the urine of diabetic db/db mice and salt-loaded hypertensive diabetic db/db mice (A) Nanoparticle tracking analysis of small uEVs isolated from urine samples from diabetic db/db mice maintained on a normal salt diet and from urine samples from hypertensive diabetic db/db mice maintained on a high salt diet. (B) Western blot of the uEV marker CD9 from the two groups, (C) Western blot of the uEV marker caveolin-1 from the two groups, (D) Western blot of the uEV marker flotillin from the two groups, (E) Western blot of the uEV marker syntenin from the two groups, (F) transmission electron microscopy analysis of uEVs from a pooled sample (n=4) of uEVs from dbdb mice maintained on a normal salt diet (left) or uEVs from hypertensive diabetic db/db mice maintained on a high salt diet (right). 100,000x direct magnification.

White arrows indicate uEVs from each group. +CTRL refers to positive control. NS refers to normal salt diet and HS refers to high salt diet in which the mice were maintained during the study. N=4 for each group. A student t test was performed to compare the two groups. A p-value <0.05 was considered significant.

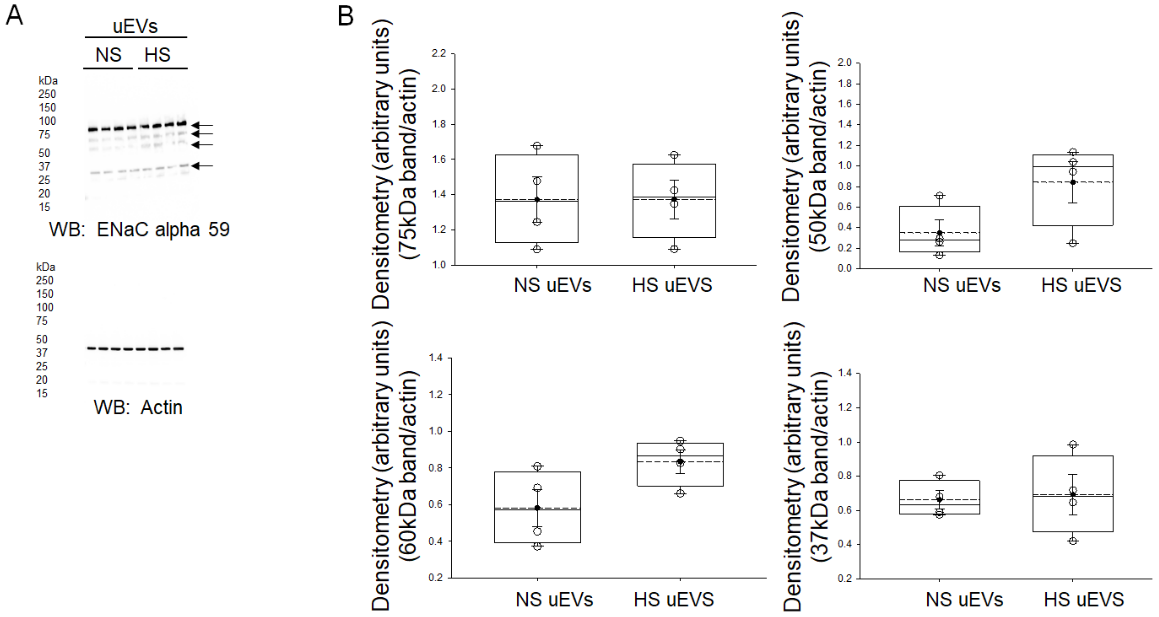

Supplementary Figure 2. Western blot and densitometric analysis of ENaC alpha protein expression in mpkCCD cells treated with small uEVs isolated from db/db mice maintained on a normal salt (NS) or high salt (HS) diet. (a) Western blot of ENaC alpha, (b) densitometric analysis of the uncleaved and cleaved forms of ENaC alpha indicated by the arrows in panel (a).

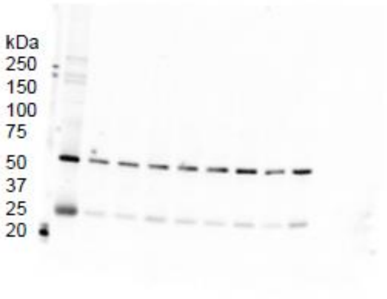

WB: CD9

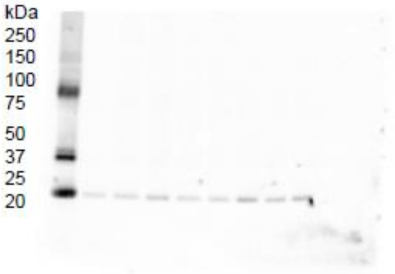

WB: Caveolin-1

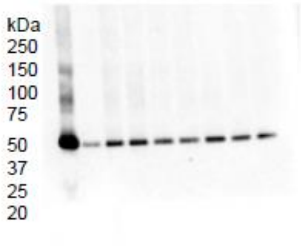

WB: Flotillin

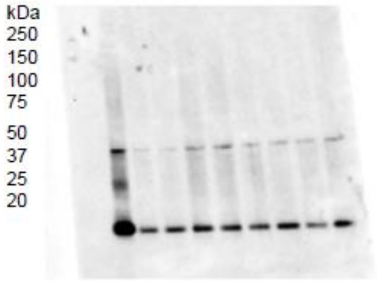

WB: syntenin

Supplementary Figure 3. Full Western blot and densitometric analysis of CD9 (Figure S1-B), Caveolin-1 (Figure S1-C), Flotillin (Figure S1-D), and syntenin (Figure S1-E).

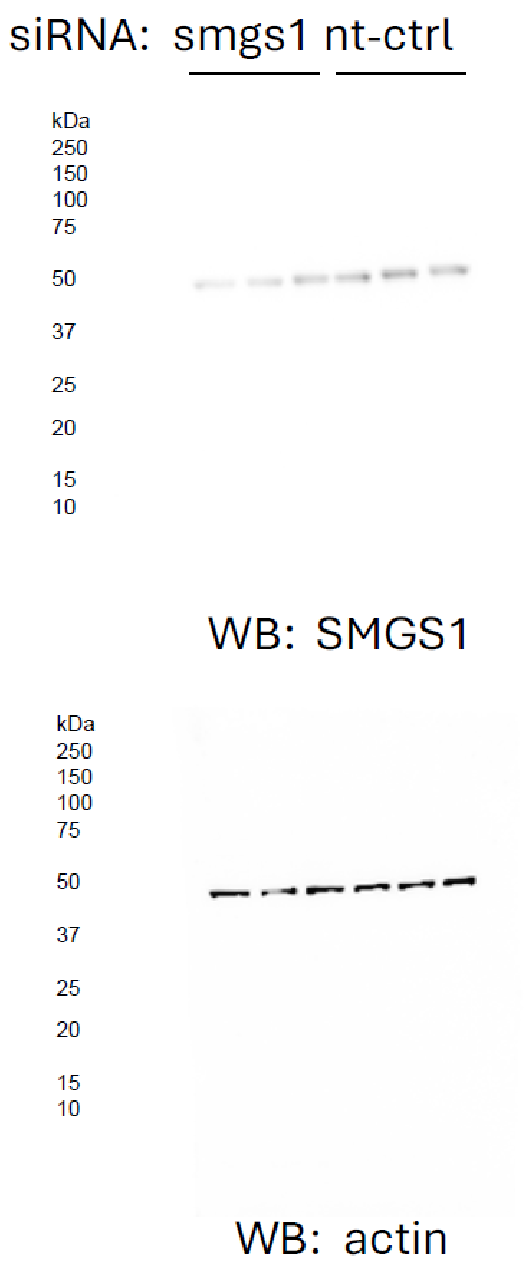

Densitometry values for Smgs1 Western blot in Figure 4F.

smg	nt	actin smg	actin nt	smg norm	nt norm
1110.87	3053.113	1710.021	2104.556	0.649624	1.450716
1368.456	4366.062	1059.142	1905.556	1.292042	2.291227
1619.113	3421.719	2085.556	2033.971	0.776346	1.682285

Supplementary Figure 4. Full Western blot and densitometric analysis ofSMGS1 and actin (Figure 4F).

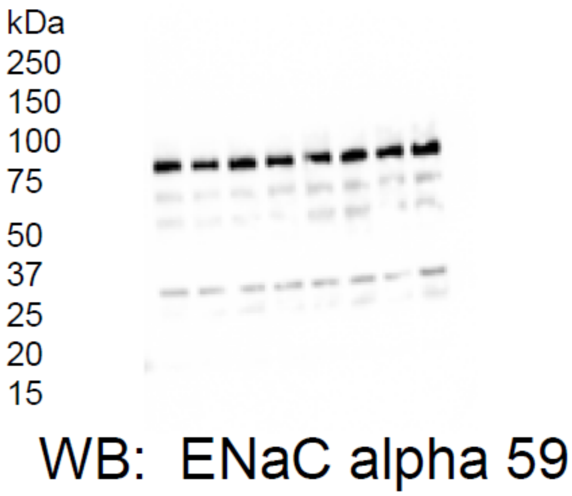

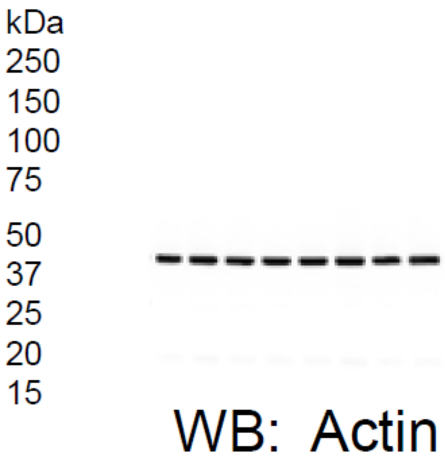

Supplementary Figure 5. Full Western blot and densitometric analysis of ENaC alpha 59 and Actin.
